# Hygrothermal Ageing of Glass and Carbon Fibre Composites Manufactured Using Different Resin Systems

**DOI:** 10.3390/polym18060696

**Published:** 2026-03-12

**Authors:** Zaneta Senselova, Allan Manalo, Abdullah Iftikhar, Omar Alajarmeh, Saya Ramakrishnan, Hiroki Sakuraba, Kate Nguyen, Brahim Benmokrane

**Affiliations:** 1 Centre for Future Materials, University of Southern Queensland, Toowoomba, QLD 4350, Australia; allan.manalo@unisq.edu.au (A.M.); abdullah.iftikhar@unisq.edu.au (A.I.); omar.alajarmeh@unisq.edu.au (O.A.); saya.ramakrishnan@unisq.edu.au (S.R.); hiroki.sakuraba@unisq.edu.au (H.S.); 2 College of Engineering, United Arab Emirates University, Abu Dhabi P.O. Box 15551, United Arab Emirates; 3 Public Works Research Institute, Tsukuba 305-8516, Ibaraki, Japan; 4 School of Engineering, Royal Melbourne Institute of Technology University, GPO Box 2476, Melbourne, VIC 3001, Australia; kate.nguyen@rmit.edu.au; 5 Department of Civil and Building Engineering, University of Sherbrooke, Sherbrooke, QC J1K 2R1, Canada; brahim.benmokrane@usherbrooke.ca

**Keywords:** hygrothermal ageing, water absorption, hydrolysis, composite degradation, durability

## Abstract

This study investigates the degradation mechanisms of glass-fibre- and carbon-fibre-reinforced polymer (GFRP and CFRP, respectively) composites fabricated either with epoxy, vinyl-ester, or bio-epoxy resins under a hygrothermal environment. Composite laminates were manufactured using the vacuum-assisted resin infusion technique and exposed to high moisture and elevated in-service temperatures of 23 °C (room temperature), 40 °C and 60 °C for up to 125 days. Changes in the physical, microstructural, chemical and mechanical properties were then assessed. CFRP and GFRP composites showed distinct differences in their hygrothermal ageing depending on the resin system used in the manufacturing. CFRP composites consistently demonstrated higher stability than GFRP composites. Epoxy resin exhibited high resistance to water absorption and hydrolysis under hygrothermal exposure. After 125 days at 60 °C, glass/epoxy (GE) and carbon/epoxy (CE) composites retained 79.0% and 72.1% of their tensile strength and 46.9% and 72.6% of their interlaminar shear strength (ILSS), respectively. Vinyl-ester composites showed high mechanical retention, with glass/vinyl-ester (GV) and carbon/vinyl-ester (CV) retaining 70.8% and 83.1% of tensile strength and 67.5% and 80.3% of ILSS, respectively. Despite this mechanical stability, evidence of hydrolysis indicated ongoing chemical degradation of the vinyl-ester resin under prolonged hygrothermal exposure. In contrast, bio-epoxy composites exhibited relatively low overall durability. Glass/bio-epoxy (GB) retained 126.5% tensile strength and 68.8% ILSS, whereas carbon/bio-epoxy retained 61.0% tensile strength and 44.3% ILSS after 125 days at 60 °C. Overall, fibre and resin types were found to have a significant effect on the hygrothermal ageing of polymer composites.

## 1. Introduction

Extreme weather conditions, driven by global warming and associated climate changes leading to disruptive environmental stability, pose significant challenges to the construction industry, impacting the long-term durability of infrastructure. Recent studies have therefore emphasised the importance of material durability and long-term performance in civil engineering applications [1,2]. Conventional construction materials like timber, steel and concrete degrade over time and require expensive and frequent maintenance and repair, highlighting the need for sustainable, more durable alternatives. Fibre-reinforced polymer (FRP) composites, known for their lightweight and high-strength properties, are becoming a viable option for industries pursuing resilient and environmentally sustainable alternatives. Because of these advantages, FRP composites are now commonly used to build, rehabilitate and retrofit existing structures, including road bridges [3,4,5], railway sleepers [6,7], electric poles [8] and building facades [9]. In these applications, FRP composites are typically exposed to severe environmental conditions, such as elevated service temperatures, high humidity, solar ultraviolet (UV) radiation, and a coastal environment. Exposure to moisture and elevated temperature is a critical factor for degradation in FRP composites, leading to time-dependent changes in physical, mechanical, and thermomechanical properties. Hygrothermal ageing promotes water diffusion into the polymer matrix and fibre–matrix interphase, where it can induce plasticisation, swelling, chain scission, and interfacial weakening [10]. These degradation mechanisms are particularly prominent in FRP composites used for long-term applications, where coupled environmental effects accelerate the deterioration. Despite their widespread use, the durability of FRP composites under combined moisture and thermal exposure remains a key limitation to developing reliable long-term performance predictions.

Available studies have demonstrated that the hygrothermal durability of FRP composites is influenced by several factors, including fibre type and volume [11,12,13], resin matrix type [14,15,16,17], and fibre–matrix interface [11,12,13,18]. The moisture absorption in FRP composites has been shown to induce plasticisation (reversible degradation) of the polymer matrix, accompanied by hydrolysis (irreversible degradation) at extended exposure times or elevated temperatures [10]. For instance, a study by Ray and Rathore [19] highlighted that the hydrophobic nature of resin-rich surfaces and the complex degradation mechanisms are the primary factors affecting mechanical properties in polymeric composites under varied environmental conditions. Mayandi et al. [20] emphasised the importance of durability in hybrid composites over their service life. They proposed methods to enhance longevity and resistance to environmental degradation, such as adding reinforcing nanomaterials or nanoparticles. In another study, Hota et al. [21] indicated that high pH (13) and high temperature (71 °C) can lead to up to 30% loss in the interlaminar shear strength of glass FRP (GFRP) composites. The long-term performance of GFRP composites with an epoxy-based particulate-filled resin coating under hygrothermal conditions was studied by Manalo et al. [22]. The authors found that, in comparison to uncoated laminates, the resin coating reduced moisture absorption and limited strength losses by up to 20% after 3000 h of exposure. The results also highlighted the coating’s protective role against resin degradation and strength reduction. Rocha et al. [23] investigated glass/epoxy composites used in wind turbine blades under hygrothermal ageing. Prolonged exposure caused significant reductions in interlaminar shear strength, indicating that moisture-induced matrix plasticisation and fibre–matrix interfacial damage are the dominant mechanisms governing mechanical deterioration under hygrothermal conditions. An investigation on the hygrothermal ageing of carbon/epoxy composites by Fan-Niu et al. [24] emphasised that moisture diffusion evolves at the fibre–matrix interface. Ageing initially reduces bending and interlaminar shear strength due to interface expansion, then temporarily improves mechanical properties through secondary curing. In the final stage, moisture saturation causes resin swelling and plasticisation, leading to strength deterioration.

Epoxy and vinyl-ester are among the most widely used thermoset matrix resins in polymers, and to the authors’ best knowledge, the long-term hygrothermal degradation behaviour of vinyl-ester composites has received less attention compared to epoxy composites. In parallel, bio-based epoxy composites are increasingly proposed as sustainable alternatives, yet their degradation mechanisms under hygrothermal ageing are poorly understood, particularly when combined with synthetic reinforcements such as glass and carbon fibres. A previous study [25] on carbon/bio-epoxy composites investigated the effect of hygrothermal ageing on interlaminar shear strength. Bio-composite specimens were conditioned at 70 °C and 85% relative humidity until moisture saturation (7 weeks). The results indicated minimal degradation, as water absorption caused only a slight reduction in interlaminar shear strength (ILSS), demonstrating good resistance to hygrothermal ageing. Critically, there is no unified experimental framework that enables direct comparison of hygrothermal degradation mechanisms between the most frequently used fibres in civil engineering, such as glass, carbon, and resin systems (epoxy, vinyl-ester, and bio-epoxy) [26]. This lack of comparative data limits the ability to generalise durability behaviour and undermines confident material selection for long-term and high-performance applications of FRP composites.

Concerning these research gaps, the present study conducts a comprehensive and systematic investigation on hygrothermal ageing in GFRP and carbon FRP (CFRP) composites manufactured using either epoxy, vinyl-ester and bio-epoxy resin. Unlike most previous studies, which focus on single fibre–resin systems or individual performance parameters, this work applies an extensive and unified conditioning and characterisation framework to enable direct comparison of the physical, mechanical and thermo-mechanical properties as well as microstructure across multiple material combinations under identical exposure conditions. The results of this study provide a better understanding of the effect of different types of fibre and resin systems in the hygrothermal ageing of FRP composites and generate additional experimental evidence to support durability-based material selection and the development of performance prediction of high-performance composite materials in aggressive environmental conditions.

## 2. Methodology

### 2.1. Materials

Glass and carbon fibres are considered in this study because of their excellent mechanical properties and durability, and for being the most used fibres in the construction industry. The fibres employed are in the form of fabric with unidirectional orientation, supplied by Colan Products Pty Limited, Huntingwood, NSW, Australia. The characteristics of glass and carbon fibres, as studied by Iftikhar et al. [27], are summarised in Table 1.

Three thermosetting resin systems, epoxy, vinyl-ester and bio-epoxy, were selected to compare their compatibility and performance with different fibre types during hygrothermal ageing, with their properties characterised in a previous study [28] and summarised in Table 2. The PrimeTM 27 epoxy resin supplied by ATL composites, Molendinar, Queensland, Australia, was selected due to its widespread application in high-performance composites, offering excellent mechanical properties and chemical resistance. The SPV6036 vinyl-ester resin supplied by Allnex, Wacol, Queensland, Australia, was chosen due to its resistance to high humidity and corrosive environments. The CCBE bio-epoxy resin, provided by Change Climate Pty Ltd., Adelaide, South Australia, Australia, was chosen as a sustainable alternative, aligning with the growing demand for environmentally friendly composite materials. Examining these resin systems alongside carbon and glass fibres enables a comprehensive comparison of durability and degradation mechanisms under hygrothermal conditions.

**Figure 1:**
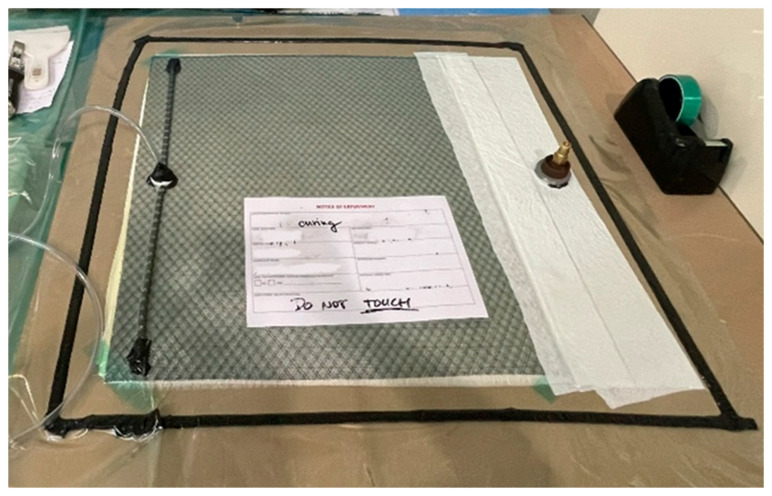
Vacuum-assisted resin infusion (VARI) method.

Composite laminates were manufactured using all combinations of the two fibre types and three resin systems, producing six distinct fibre–matrix configurations. All laminates were fabricated using the vacuum-assisted resin infusion (VARI) method with ten layers of unidirectional fabric, resulting in panels approximately 2 mm thick, as shown in Figure 1. The VARI process ensured consistent resin impregnation and resulted in relatively controlled fibre volume fractions across all composites.

The curing process was selected in accordance with the manufacturer’s recommendations for each resin system. The epoxy resin was cured for 24 h at room temperature under vacuum, followed by post-curing at 50 °C for 16 h. The vinyl-ester resin was cured for 24 h at room temperature under vacuum and subsequently post-cured at 120 °C for 2 h. The bio-epoxy resin required a longer curing period and was cured at room temperature for 7 days, with vacuum applied for the first 3 days, followed by post-curing at 80 °C for 4 h.

### 2.2. Test Specimens

Test specimens were cut from the manufactured composite panels before conditioning using a water jet. For each composite type, conditioning temperature, and exposure duration, five specimens were cut for tensile and interlaminar shear strength characterisation, two specimens for dynamic mechanical analysis, and two samples each for non-destructive testing, such as FTIR, water absorption, DSC, and microscopic observation. A total of 50 specimens for tensile testing, 50 specimens for interlaminar shear testing, 20 specimens for DMA testing and 10 specimens for chemical, physical and microstructural analysis were cut from composite panels for each fibre and resin combination, as summarised in Table 3.

### 2.3. Hygrothermal Conditioning

The FRP composites were subjected to an elevated in-service temperature and high moisture to simulate the effect of accelerated ageing in a hygrothermal environment. All specimens were fully immersed in distilled water in a glass container with lids. These containers were placed in a Vötsch Technil, Balinden, Germany, environmental chamber to maintain precise temperature and relative humidity conditions. The ageing process was conducted over extended durations of 42, 83 and 125 days (approximately 1000, 2000 and 3000 h, respectively) at three selected temperatures: room temperature (23 °C), 40 °C, and 60 °C. The selected exposure durations represent staged intervals to capture early-, mid-, and late-stage moisture absorption and associated physical, mechanical, thermo-mechanical and microstructural changes. These temperature levels were specifically chosen to represent (i) ambient service conditions (23 °C), (ii) moderately elevated in-service conditions (40 °C), and (iii) accelerated ageing conditions (60 °C) to increase moisture diffusion and degradation mechanisms. All temperatures were maintained below the glass transition temperature (Tg) of the resin systems, as determined by Iftikhar et al. [27], to ensure that the FRP composites remained in a solid-state condition without undergoing thermal softening or phase transitions during the conditioning process. This approach allows for the evaluation and direct comparison of long-term durability and moisture-induced degradation mechanisms without interference from thermal deformation. Moreover, these ageing temperatures and exposure durations are often used by previous researchers, which enable a direct comparison of their findings with the results of this study.

The level of water in glass containers was monitored and replenished as needed at least once a week to ensure that samples remained fully submerged for the entirety of the conditioning period. Water absorption was measured throughout the entire conditioning period by periodically removing the samples from the container with water, gently wiping their surface with a paper towel to remove excess surface moisture, and immediately recording their mass. After weighing, the samples were returned to the container with water to continue exposure. The test method proposed in ASTM D570-22 [33] was used for measuring water absorption. The water absorption data provides insight into the diffusion behaviour and potential degradation mechanisms of the materials under hygrothermal ageing conditions, which are relevant to real-world service environments.

After hygrothermal ageing, specimens were removed from the water, dried at room temperature, and tested within a maximum period of one week.

### 2.4. Test Methods and Procedures

The characterisation of composite materials subjected to hygrothermal ageing was carried out according to relevant ASTM standards. A combination of thermal, mechanical, and chemical characterisation techniques was employed to comprehensively evaluate the effects of hygrothermal ageing on the composite materials investigated in this study.

Differential Scanning Calorimetry (DSC) was used to assess the curing behaviour and degree of cure of the matrix system, in accordance with ASTM D3418-21 [32]. Uncured and cured resin samples (5–10 mg) were analysed at a heating rate of 20 °C/min. The degree of cure was calculated by comparing the residual exothermic heat of the cured samples with the total heat of reaction of the uncured resin, using the area under the exothermic peak.

**Figure 2:**
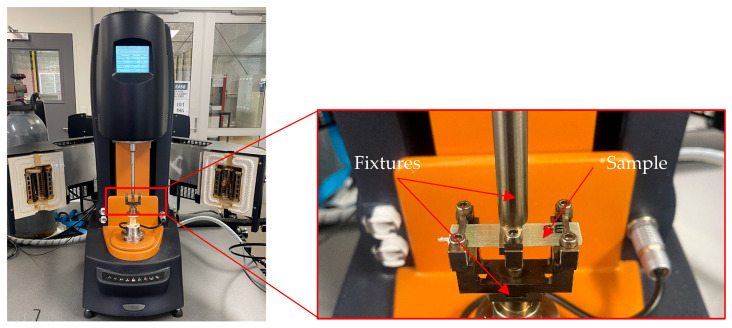
TA Instruments Hybrid Rheometer DMA set-up.

Dynamic mechanical analysis (DMA) was conducted to determine the glass transition temperature (Tg) of both unaged and hygrothermally aged samples, allowing for the assessment of changes in properties due to ageing. Testing was performed using a TA Instruments Hybrid Rheometer, New Castle, Delaware, USA, in accordance with ASTM D7028-07 [31], with rectangular specimens of 8–10 mm width and 45 mm length, as shown in Figure 2. In DMA, the samples were subjected to small oscillatory deformation while being heated, enabling measurement of the storage and loss modulus as a function of temperature. The temperature was increased from room temperature to 150 °C for epoxy, 180 °C for vinyl-ester and 100 °C for bio-epoxy at a constant heating rate of 5 °C/min. The glass transition temperature was identified from the peak of the tan δ curve.

Fourier Transform Infrared Spectroscopy (FTIR) was performed using a Nicolet iS50-FT-IR, Thermo Fisher Scientific, Waltham, MA, USA, to assess the chemical composition and possible changes in the polymer matrix. The analysis was done in ATR (Attenuated Total Reflectance) mode, which allows the infrared light to interact with just the surface of the sample without needing to prepare very thin slices. Spectra were recorded over the range of 4000–1500 cm^−1^, focusing on the main functional groups relevant for polymer degradation. The fingerprint region below 1500 cm^−1^ was not considered because the degradation analysis focused on changes in functional-group bands in the higher wavenumber region, while the fingerprint region contains complex overlapping bands that are less directly interpretable for assessing moisture-induced and hydrolytic degradation in these polymers. By comparing the spectra before and after ageing, changes in peak positions and intensities were used to identify any chemical changes or degradation in the resin.

Microstructural analysis of the composites was carried out using an Olympus DSX100 digital microscope, Evident, Macquarie Park, New South Wales, Australia, enabling detailed surface and internal morphology evaluation. This allowed for the visualisation of fibre distribution, matrix uniformity, and potential defects such as voids, fibre pull-out, or delamination. Observations from the microscope provided insights into the effects of hygrothermal ageing on the microstructure properties. Micrographs were acquired using optical magnification of up to 50×. No surface coating or chemical treatment was applied prior to the observation. Specimens were examined in the as-conditioned state, with only the specimen edges polished to enable through-thickness microstructural observation.

**Figure 3:**
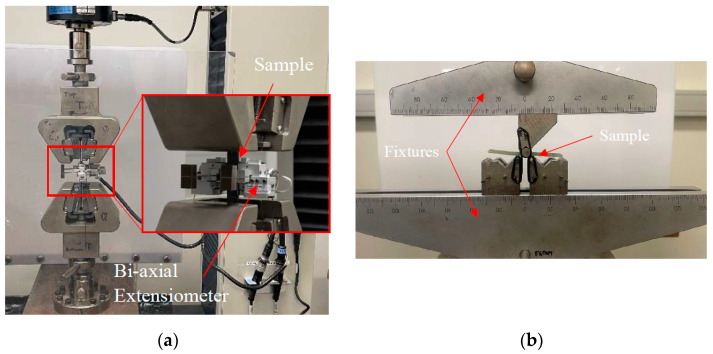
(**a**) Tensile test set-up; (**b**) ILSS test set-up.

Mechanical testing, including tensile and interlaminar shear strength tests, was performed using an MTS 100kN universal testing machine, MTS Systems, Eden Prairie, Minnesotta, USA. Tensile specimens were prepared in a dog-bone shape in accordance with ASTM D3039/D3039-M [29], and testing was carried out at a crosshead speed of 1.3 mm/min. This allowed assessment of the longitudinal stiffness and strength of the composites, providing insight into fibre load-bearing capacity and the overall structural integrity of the composites. Tabs were applied to the ends of the tensile specimens to improve load transfer and prevent premature failure at the gripping areas. The tabs were bonded to both ends of each specimen to ensure a stronger and more uniform grip compared to the central gauge section. Composite tabs were used to maintain material compatibility and reduce stress concentration at the interface for glass-fibre-reinforced composites. In contrast, metal tabs were applied to carbon-fibre-reinforced specimens to provide higher stiffness and more reliable clamping during testing. Elongation of composites during testing was measured using a bi-axial extensometer, as shown in Figure 3. The modulus of elasticity was determined from the initial linear region of 1000–3000 μm of the stress–strain curve using an extensometer.

Interlaminar shear strength (ILSS) testing was performed to evaluate the interfacial integrity and shear load-bearing capacity of the composite laminates. Specimens were prepared according to ASTM D2344/D2344M-00 [30] and tested in a three-point bending configuration, with a span of 10 mm, resulting in a shear span-to-depth ratio of 5. This short-span set-up ensures that failure occurs predominantly in the interlaminar region rather than in the fibre-dominated areas, allowing accurate assessment of the matrix–fibre interface. Tests were conducted at a crosshead speed of 1 mm/min, and the maximum load was used to calculate the ILSS. Observing ILSS behaviour provides insight into the effects of hygrothermal ageing on interfacial strength.

The coefficient of variation (CoV) was calculated for all mechanical properties to quantify data dispersion and assess experimental repeatability.

## 3. Results and Discussion

### 3.1. Effect on the Degree of Cure

The degree of cure (*DoC*) was determined using Differential Scanning Calorimetry (DSC) to assess the curing behaviour of FRP composites with different resin systems. Measurements were conducted on both uncured resin samples (to determine the total heat of reaction, Δ*H_t_*) and fully cured FRP composites (to determine the residual heat of reaction, Δ*H_r_*). The *DoC* was calculated using Equation (1):

(1) $$DoC\left(\%\right)=\left[1-\left(\frac{{∆H}_{r}}{{∆H}_{t}}\right)\right]\times 100,$$

The calculated *DoC* values for all composite systems are summarised in Table 4, showing the influence of resin type and fibre reinforcement on curing efficiency. All samples exhibited a *DoC* of above 99%, indicating that the FRP composites were almost fully cured and that the hygrothermal exposure did not affect the degree of cure.

The fibre volume fraction and resin content of the FRP composite laminates were determined using a burn-out test, following ASTM D3171-22 [34]. Specimens of size 60 mm × 80 mm and weighing approximately 18–20 g were heated in a muffle furnace at 580 °C (±50) for 3 h to thermally decompose and combust the polymer matrix, leaving the reinforcing fibres intact. The remaining fibres were weighed, and the fibre weight fraction was calculated based on initial and final mass. Using the fibre weight fraction and known densities of the fibres and matrix presented in Table 1 and Table 2, respectively, the fibre volume fraction was then calculated. These values are presented in Table 4. The composites exhibited a higher content of fibre than resin by weight, contributing to their overall strength and stiffness. Carbon fibre composites showed the highest volume fraction, reaching up to 61.05%, which corresponds to their superior stiffness and strength. Composites with vinyl-ester resin showed higher fibre content than other resins, which indicates increased strength and stiffness, but potentially reduced toughness due to the lower resin content. In contrast, bio-epoxy composites had the lowest fibre volume fractions, resulting in more resin-rich areas that may influence flexibility and long-term performance under hygrothermal conditions.

### 3.2. Physical Degradation

#### 3.2.1. Change of Colour

**Figure 4:**
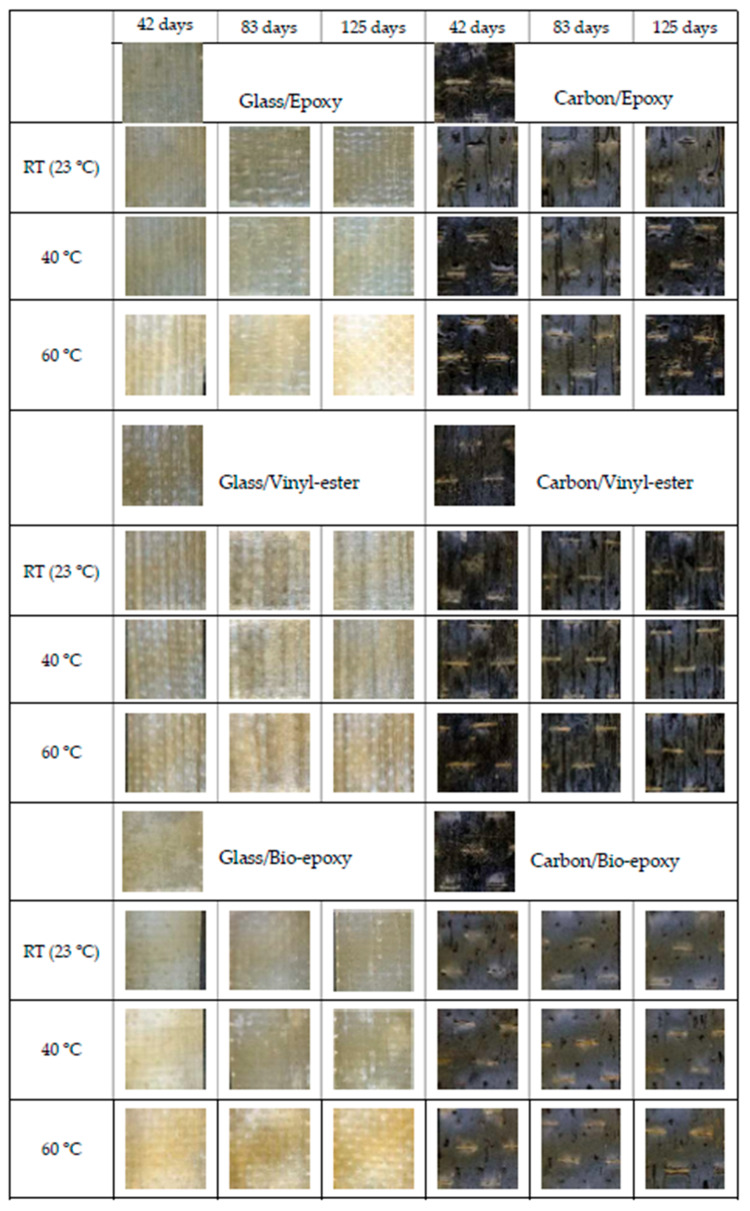
Change of colour in FRP composites.

Figure 4 shows the change in physical appearance of FRP composites during accelerated ageing. As can be seen, the carbon fibre composite did not exhibit any noticeable discolouration under any of the tested temperatures or exposure durations. Only the bio-epoxy-based composites showed a slightly more matte surface after ageing, suggesting minor alterations in appearance.

##### Fibre Type Effect on Change of Colour

This is primarily due to the intrinsic black colour of carbon fibres, which makes visual detection of colour change difficult. Similar observations have been reported in ageing studies of carbon fibre composites by [35], where the dark surface masked colour transitions despite underlying matrix alterations. However, the overall black colour of the laminate prevented any significant or clearly observable discolouration. In contrast, the glass fibre composites exhibited more pronounced changes across all resin types.

##### Resin Type Effect on Change of Colour

The glass fibre composites manufactured with epoxy resin displayed clear discolouration at 60 °C for all ageing durations (42, 83, and 125 days). No noticeable discolouration was observed at room temperature or at 40 °C for a shorter duration. The first signs of discolouration at 40 °C appeared only after 125 days of ageing. While the unconditioned samples were relatively translucent, the aged samples—particularly those exposed to higher temperatures and longer durations—lost this transparency and exhibited a more noticeable whitening.

The glass fibre composites with vinyl-ester resin were more resistant to colour change. Before conditioning, samples were mostly translucent, with only a very faint natural shade. Only slight yellowing was observed at 60 °C, and even then, the extent of discolouration was visible but limited, and far less pronounced than that observed in the epoxy-based composites. This behaviour is consistent with the previous study [28] reporting superior yellowing resistance of vinyl-ester systems compared with epoxy matrices.

On the other hand, the glass fibre composites with bio-epoxy resin showed the most significant discolouration. Pronounced colour changes were evident at 60 °C for all exposure durations, and visible discolouration also occurred at 40 °C after 42 days. As expected, increasing both the temperature and the ageing duration intensified the degree of colour change in this resin system.

The most pronounced discolouration/yellowing of bio-epoxy GFRP aligns with its higher water absorption compared to vinyl-ester GFRP, which exhibited the lowest water absorption, indicating greater physical degradation (Table 5). Higher water absorption accelerated chemical changes in the matrix, resulting in more noticeable discolouration [36].

Overall, the observed discolouration trends indicate that fibre type governs the visibility of colour change, while resin type controls its severity, with bio-epoxy GFRP showing the greatest yellowing and vinyl-ester GFRP the highest colour stability. This trend is consistent with the findings reported by Iftikhar et al. [28], who observed the highest yellowness in bio-epoxy composites, followed by epoxy, with vinyl-ester showing the greatest colour stability under accelerated ageing.

#### 3.2.2. Effect on Water Absorption

The water absorption behaviour of FRP composites can be a basis for evaluating their long-term durability and performance under a hygrothermal environment. The water uptake of FRP composites can cause plasticisation of the polymer matrix, debonding at the fibre–matrix interface, microcracking, and other degradation mechanisms that affect the mechanical and thermal properties of composite materials. In the tested specimens, this was reflected in more pronounced discolouration and surface changes observed in bio-epoxy composites, while vinyl-ester composites showed limited visual and physical degradation due to lower water absorption. Carbon fibre composites, although absorbing more water than glass-based composites, showed negligible visible discolouration due to the dark colour of the fibres, though minor surface changes could still be detected. Water uptake in ideal composites (void-free) is dominated by diffusion, but in most manufactured composites, interfacial regions, defects (voids, microcracks), and fibre–matrix interfaces play a major role, helping to explain the differences in behaviour observed among the investigated FRP composites.

Rectangular specimens (20 × 10 mm) were fully immersed in water at three different temperatures, room temperature (23 °C), 40 °C, and 60 °C, to quantify water absorption, as in ASTM D570-22 [33]. Samples were removed from the water and dried with a paper towel to remove surface moisture and weighed at frequent periods on a balance with a maximum capacity of 200 g and an accuracy of 0.0001 g. To calculate mass change, the following equation was used:

(2) $$Mass\;change\%=\frac{m_{t}-m_{i}}{m_{t}}\times 100,$$

where *m_t_* is the sample mass at time *t* and *m_i_* is the initial sample mass.

For each combination, three samples were measured, and the average data were reported.

**Figure 5:**
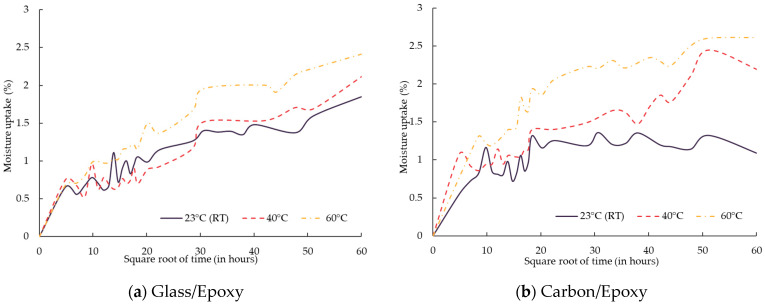

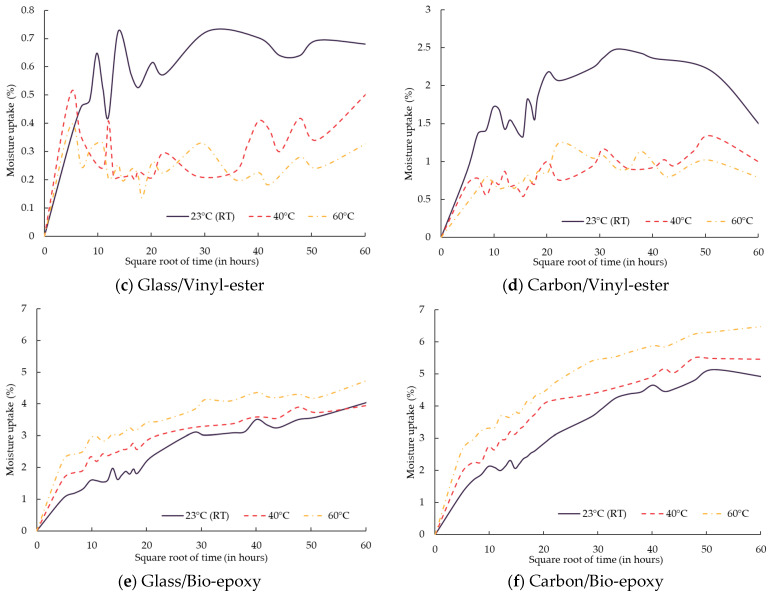
Water uptake of composites.

Figure 5 represents the average water absorption plotted against the square root of immersion time (in hours) for specimens conditioned at room temperature (23 °C), 40 °C and 60 °C, where each data point represents the mean of three specimens.

##### Fibre Type Effect on Water Absorption

Comparison of the moisture uptake of the investigated FRP composites is summarised in Table 5. The results indicated that carbon FRP composites exhibited higher water absorption than glass FRP composites. The higher fibre volume fraction in the carbon FRP compared to glass FRP composites may have resulted in increased voids or poor matrix impregnation, providing a pathway for water to diffuse more rapidly. These factors may promote interfacial debonding, providing additional pathways for moisture uptake.

##### Resin Type Effect on Water Absorption

Increasing the conditioning temperature results in higher moisture uptake for both the epoxy and bio-epoxy resins, in contrast to the vinyl-ester resin, where moisture absorption decreases as temperature increases. A similar observation was noted by Yin et al. [17], where the moisture uptake of vinyl-ester was slightly higher at lower temperatures than at 60 °C. This behaviour can be attributed to the fact that at room temperature, vinyl-ester primarily absorbs moisture through diffusion and network relaxation, resulting in a higher moisture uptake, whereas at 60 °C, additional processes such as hydrolysis and leaching may occur, potentially causing slight resin mass loss and reducing the net water uptake [17]. Vinyl-ester composites exhibited a clear moisture saturation behaviour within the exposure duration, with the saturation time strongly dependent on fibre type and conditioning temperature. For glass/vinyl-ester composites, saturation was reached after approximately 120 h at room temperature. Increasing the temperature to 40 °C and 60 °C significantly accelerated the process, with saturation occurring at around 50 h. In contrast, carbon/vinyl-ester composites required a substantially longer time to reach saturation, occurring at approximately 400 h at room temperature and around 120 h at 40 °C and 60 °C. However, epoxy and bio-epoxy composites did not exhibit a distinct saturation plateau within the investigated exposure duration, which indicates continuous moisture uptake and suggests ongoing degradation processes.

Among all combinations of FRP composites, the highest water uptake was observed for the carbon/bio-epoxy and glass/bio-epoxy composite, with water uptakes of 6.51% and 4.81%, respectively. Significantly higher water absorption of bio-epoxy resin compared to epoxy and vinyl-ester resin was also noted by Iftikhar et al. [28]. This behaviour of bio-resin was attributed to the presence of hydrophilic functional groups. Additionally, the glycerol core in bio-epoxy, with its three hydroxyl groups, promotes hydrogen bonding with water, further enhancing hygroscopicity.

#### 3.2.3. Effect on Glass Transition Temperature (T_g_)

##### Fibre Type Effect on Tg

The glass transition temperature (Tg) of FRP composites before and after hygrothermal ageing, determined from DMA based on tan δ, is summarised in Table 6. The CoV values remained below 12%, demonstrating reasonable consistency of the DMA measurements. There was minimal difference between glass and carbon FRP composites; however, significant differences were observed across resin systems. The highest values of Tg of 119.3 °C and 120.8 °C were reported for vinyl-ester-based composites, whereas the bio-epoxy-based composites exhibit the lowest values of Tg of 62.1 °C and 65.1 °C. The epoxy-based composites reported values of Tg of 85.2 °C and 90.7 °C before exposure.

##### Resin Type Effect on Tg

**Figure 6:**
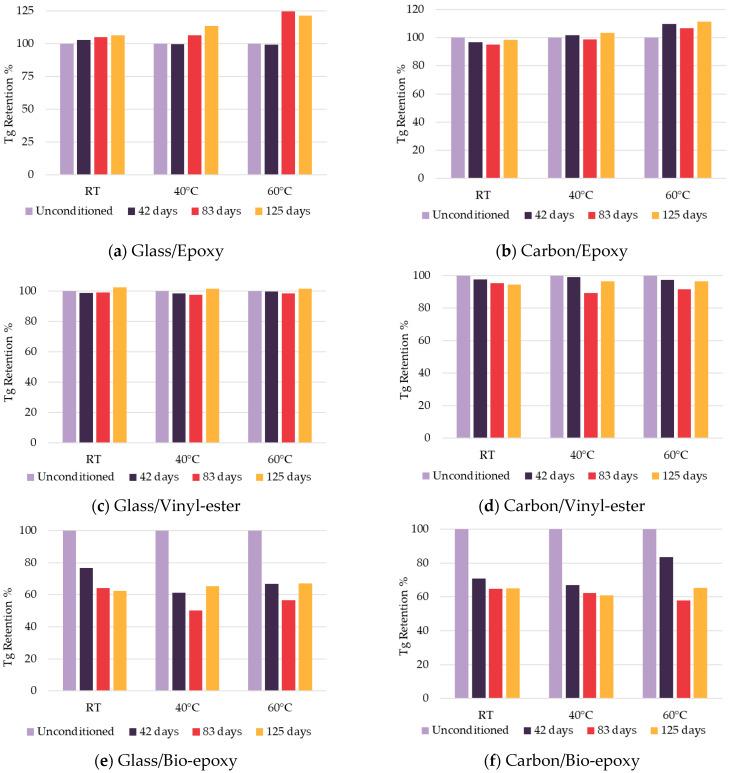
Retention of the glass transition temperature of composites.

The evolution of Tg in FRP composites subjected to long-term environmental exposure revealed significant differences among the resin systems, as shown in Figure 6. Glass/epoxy composites exhibited a progressive increase in Tg with increased exposure and temperature (Figure 6a). Similarly, carbon/epoxy composites showed Tg increases with increasing temperature and duration, especially at 40 °C and 60 °C after 42, 83 and 125 days, with a slight decrease in Tg retention at RT (Figure 6b). Progressive increases in Tg with exposure time and temperature may be associated with crosslinking effects due to residual reactive groups in the resin. This behaviour is supported by a decrease in C-H stretching intensity in FTIR spectra, along with broadening of O-H stretching peaks, suggesting additional oxidation or catalyst activation, as also explained by [37]. However, this T_g_ increase should not be interpreted as an intrinsic improvement in long-term durability, as concurrent mechanical degradation was observed. Vinyl-ester-based composites maintained high Tg retention with minor variations over time, indicating their superior thermal stability and resistance to environmental ageing, as shown in Figure 6c,d. In contrast, bio-epoxy-based composites demonstrated the most pronounced decrease in Tg. Glass/bio-epoxy composites dropped to 60–75% retention after 42 days and further decreased to 50–65% at 40 °C and 60 °C after 83 days, with slight recovery after 125 days (Figure 6e). Carbon/bio-epoxy composites followed a similar trend, with Tg retention between 55 and 85% depending on temperature and exposure duration (Figure 6f). These observations highlight the significant influence of the hydrophilic functional groups present in the bio-epoxy resin, which promote higher water absorption and accelerate hydrolytic degradation, leading to a pronounced reduction in Tg [38]. It should be noted that the initial T_g_ of the bio-epoxy composites (≈62–65 °C, Table 6) places the 60 °C conditioning near T_g_. Moisture absorption can further decrease T_g_ through plasticisation [39], such that the matrix may locally approach a viscoelastic state during exposure. The severe degradation observed at 60 °C for bio-epoxy composites should be interpreted as near-T_g_ hygrothermal ageing.

### 3.3. Mechanical Degradation

#### 3.3.1. Effect on Tensile Properties

**Figure 7:**
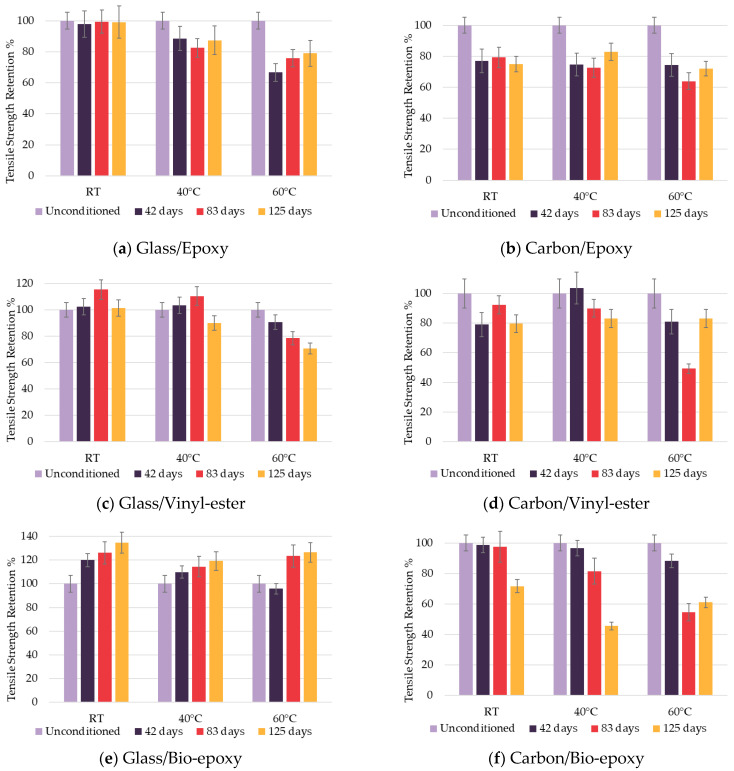
Tensile strength retention of composites.

The tensile strength test properties, as shown in Table 7, reveal that carbon FRP composites are superior to their glass-fibre-reinforced counterparts. Differences between various resins were minimal, suggesting that resin has a limited influence on initial tensile properties. However, there were no significant changes in modulus of elasticity in accelerated ageing (Table 8), compared to strength. This behaviour can be explained by the fact that the tensile strength and modulus of elasticity are governed more by the fibres than by the resin. The CoV for tensile strength remained below 14.1% and below 7.3% for modulus of elasticity, indicating acceptable repeatability of the mechanical measurements (*n* = 5). Figure 7 represents tensile strength retention after hygrothermal exposure.

##### Fibre Type Effect on Tensile Properties

**Figure 8:**
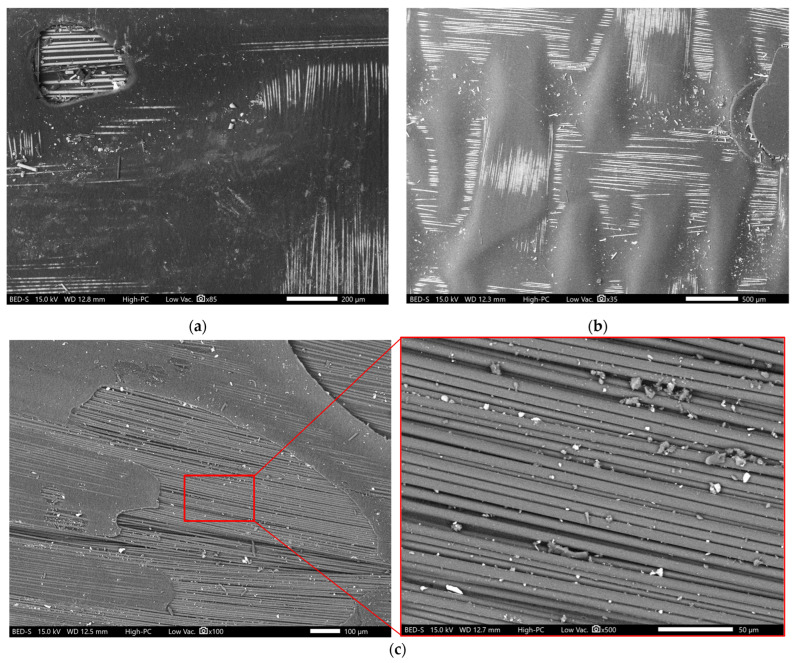
Surface morphology: (**a**) unexposed samples; (**b**) GFRP composites after exposure; (**c**) CFRP composites after exposure.

Glass FRP composites generally maintain or even increase strength over time (bio-epoxy-based composite, Figure 7e), likely due to a combination of moisture-induced matrix plasticisation, stress release mechanisms, and the influence of moisture diffusion [40], as well as internal stress effects on tensile response in epoxy-based composites [41]. In contrast, carbon FRP composites exhibited lower tensile strength retention than glass FRP composites after hygrothermal ageing. This behaviour can be attributed to resin recession at the surface, exposure of fibre bundles, and moisture-induced degradation of the surrounding matrix and fibre–matrix interface, as was noted in the samples and shown in Figure 8c, leading to weakened load transfer and stress concentration.

##### Resin Type Effect on Tensile Properties

Resin type strongly influenced degradation mechanisms. Epoxy-based composites showed the most stable strength retention for both glass and carbon fibres, as shown in Figure 7a,b. This can be caused by the dense crosslinked network and low hydrophilicity of epoxy resin, limiting moisture absorption and chemical degradation. The lowest retention for glass/epoxy was 66.7% at 60 °C after 42 days, while for carbon/epoxy, it was 63.9% at 60 °C after 83 days. After 125 days of exposure, tensile strength retention increased to 79.0% for glass and 72.1% for carbon. Vinyl-ester-based composites exhibited more variability: while glass fibre composites remained relatively stable (Figure 7c), carbon fibre composites (Figure 7d) experienced a significant decrease to 49.3%, likely due to microstructural damage such as fibre–matrix debonding under hygrothermal exposure, which compromised load transfer at intermediate conditioning durations. After 125 days of exposure, tensile strength retention reached 70.8% for glass and 83.1% for carbon composites, reflecting partial recovery or stabilisation of mechanical performance over extended ageing. Bio-epoxy showed the highest variability, with glass fibre composites showing a continuous increase in strength (Figure 7e), due to possible viscoelastic relaxation effects as a physical ageing in the matrix resin system. Tensile strength retention reached 126.5% of the initial value after 125 days of exposure. As reported by Le Guen-Geoffroy et al. [42], physical ageing in epoxy-based composites can proceed rapidly under certain conditions and may result in a temporary increase in mechanical properties due to stress relaxation and structural reorganisation within the polymer network, whereas carbon fibre composites experienced a pronounced reduction (Figure 7f), decreasing to 45.5% after 83 days of exposure at 40 °C because of accelerated moisture ingress through resin-rich areas and interfacial weakness. A partial recovery was observed after 125 days, with tensile strength retention reaching 61.0%. FTIR analysis confirmed hydrolytic degradation in vinyl-ester and bio-epoxy composites (Table 9), correlating with the observed reductions in strength, whereas epoxy composites showed no evidence of hydrolysis, consistent with their stable performance.

**Figure 9:**
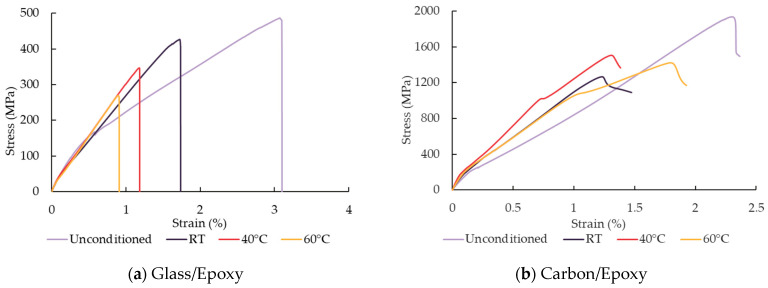

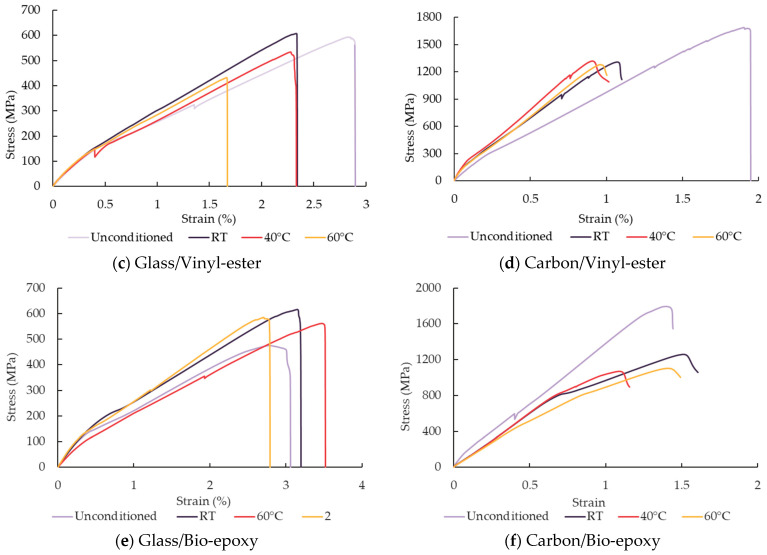
Stress–strain curve of composites.

The stress–strain curves (Figure 9) show that hygrothermal ageing primarily reduces strain at failure and changes the failure mode. In glass/epoxy, ageing leads to earlier failure and a steeper linear region, indicating reduced ductility and more brittle behaviour. Similar behaviour was observed by Iftikhar et al. [28]. In carbon/epoxy, ageing results in reduced strain at failure together with a change in stiffness and earlier deviation from linearity, consistent with matrix plasticisation reducing load-transfer efficiency while fibres remain load-bearing. This behaviour aligns with the stable FTIR O-H/C-H ratio (Table 9), confirming predominance of physical degradation. Glass/vinyl-ester exhibits moderate slope changes but a clear reduction in strain at failure at elevated temperature, suggesting interfacial weakening and hydrolytic effects, as supported by the increased O-H/C-H ratio (Table 9). In carbon/vinyl-ester, ageing similarly reduces strain capacity and introduces premature stress drops, indicating fibre-dominated fracture. In glass/bio-epoxy, curves become more linear with abrupt fracture, reflecting loss of ductility after exposure. In carbon/bio-epoxy, the reduction in strain at failure is even more pronounced, accompanied by early failure. This correlates with severe interfacial degradation and increased hydroxyl formation (Table 9).

#### 3.3.2. Effect on Interlaminar Shear Strength (ILSS)

Table 10 summarises the ILSS of FRP composites before and after hygrothermal conditioning. ILSS results for unconditioned samples revealed that the lowest values were observed in the bio-epoxy composites. For instance, the ILSS reported for glass and carbon composites was 23.36 MPa and 37.51 MPa, respectively. In contrast, both epoxy, 41.95 MPa and 41.62 MPa, and vinyl-ester, 40.39 MPa and 40.52 MPa, systems exhibited relatively high ILSS values regardless of the type of fibres used, indicating minimal influence of fibre type. However, for the bio-epoxy composites, a significant difference was noted depending on the fibre type. Carbon fibre composites showed increased ILSS by nearly 15 MPa compared to glass fibre composites, as given in Table 10. The CoV for ILSS remained below 10.3% (*n* = 5), indicating good consistency of the interlaminar shear measurements.

**Figure 10:**
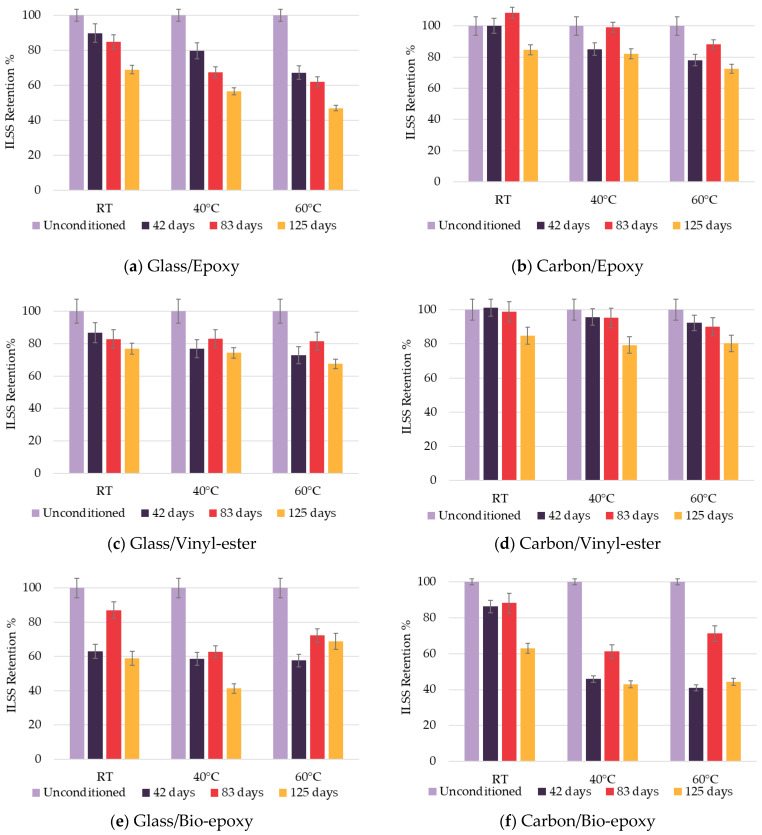
ILSS retention of composites.

ILSS retention of composite materials is shown in Figure 10. Degradation of ILSS was observed for all composite combinations.

##### Fibre Type Effect on ILSS

Carbon fibre composites performed better after exposure than their glass fibre counterpart, except for the bio-epoxy composites. This is in contrast with the previous study [25], which reported minimal ILSS degradation on bio-composites under hygrothermal conditions. The extent of ILSS degradation is therefore a combined effect of fibre type, which determines interfacial bonding strength, and resin type, which governs hydrophilicity and susceptibility to hydrolysis. In CFRP composites, carbon fibres are typically coated with epoxy sizing, which ensures strong adhesion with the matrix and helps preserve interlaminar shear performance despite surface apparent matrix loss and interfacial debonding [24,27]. In contrast, glass fibres are often treated with silane-based (si-O-Si) sizing, which is susceptible to hydrolytic degradation under hygrothermal conditions, potentially reducing fibre–matrix adhesion and ILSS retention [28]. For carbon fibre composites, the pronounced matrix loss and interfacial debonding exposed portions of the fibre bundles, further reducing ILSS (Figure 8). These findings highlight that the fibre–matrix interface plays a critical role in load transfer between layers, and its degradation directly contributes to lower ILSS retention after hygrothermal exposure.

##### Resin Type Effect on ILSS

The degradation of ILSS in carbon/bio-epoxy composites can be explained by the presence of resin-rich areas in bio-epoxy composites, which promote higher water absorption as observed in Figure 5. This effect is associated with matrix swelling and chemical changes, including enhanced hydrolytic reactions, which can weaken the fibre–matrix interface and compromise load transfer between layers, thereby affecting ILSS. The highest degradation was measured for bio-epoxy-based composites. This agrees with the literature [39] showing that moisture ingress weakens the fibre–matrix interface and plasticises the resin, reducing shear strength in aged composites.

ILSS retention of epoxy-based composite exhibited a consistent and gradual decline with increasing conditioning duration and temperature, consistent with the study [23] of progressive ILSS loss in epoxy resins under hygrothermal ageing due to matrix softening. Strength retention of epoxy composites reached 46.9% and 72.6% after 125 days at 60 °C for glass and carbon composites, respectively.

Vinyl-ester composites showed moderate degradation, with 67.5% and 80.3% ILSS retention after 125 days at 60 °C for glass and carbon composites, respectively. As shown in a study [10], vinyl-ester resins are less hydrophilic than epoxies, leading to intermediate ILSS loss.

**Figure 11:**
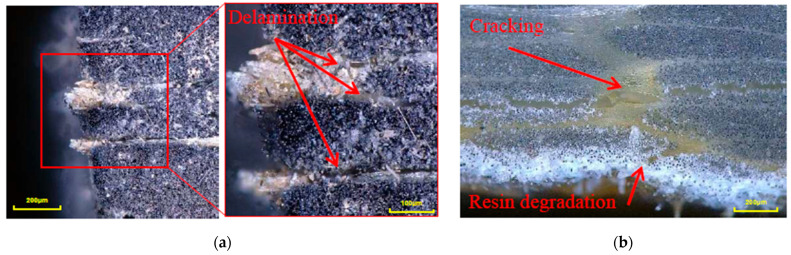
Delamination and resin degradation of bio-epoxy composites after exposure, observed at the specimen edge (**a**) CFRP, (**b**) GFRP. Red arrows indicate resin degradation, delamination and cracking, red box highlights the location from which magnified micrographs were obtained.

Bio-epoxy composites exhibited a non-monotonic trend of ILSS retention, with slightly higher retention after 83 days of exposure compared to earlier exposure, followed by a decrease in retention after 125 days, with strength retention of 68.8% and 44.3% for glass and carbon composites, respectively. The observed short-term increase in ILSS retention may be associated with physical effects such as matrix plasticisation and viscoelastic stress relaxation, as evidenced by the decreased Tg after exposure of 83 days, or moisture equilibrium within the resin, leading to temporary modification of the interfacial bond, consistent with observations reported for epoxy-based composites under hygrothermal ageing [42]. This apparent short-term increase should therefore be interpreted with caution and does not necessarily indicate intrinsic strengthening. However, prolonged exposure activated degradation mechanisms, including hydrolysis (observed in vinyl-ester and bio-epoxy composites, Table 9), fibre–matrix debonding, delamination and microcracking (Figure 11).

Overall, the results demonstrate that composites with strong fibre–matrix adhesion (carbon) and low matrix hydrophilicity (epoxy) exhibit higher ILSS retention, whereas bio-epoxy and glass fibres are more vulnerable due to interface weakening and hydrolytic degradation. Moreover, the ILSS retention is relatively lower than the tensile strength retention of the FRP composites, regardless of the fibre type. This indicates that ILSS is highly sensitive to fibre–matrix adhesion and type of resin system, which should be considered in composite manufacturing and applications.

### 3.4. Chemical Degradation

**Figure 12:**
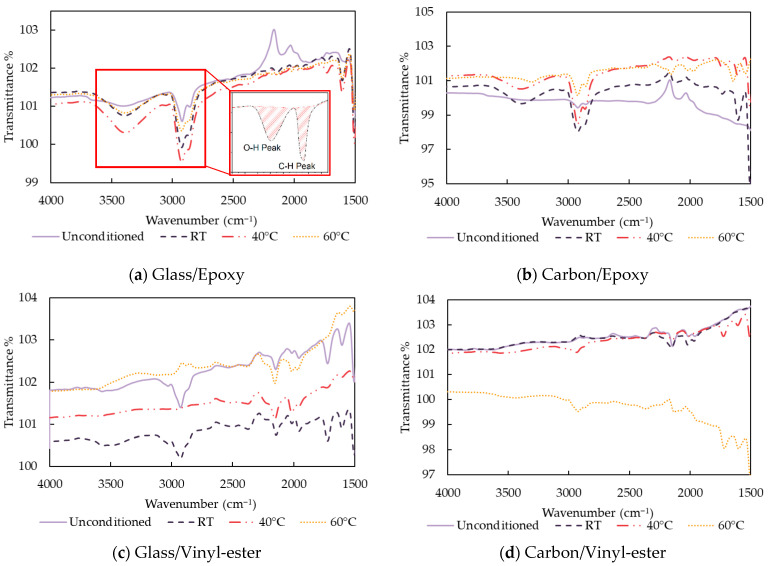

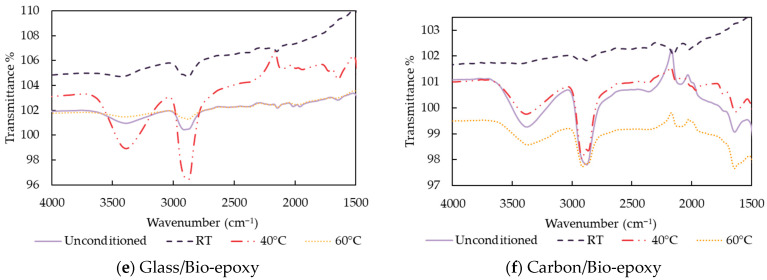
FTIR of unconditioned and conditioned samples of composites after 125 days.

Figure 12 shows the measured absorption of infrared light at different wavelengths using Fourier Transform Infrared Spectroscopy (FTIR) for the FRP composites considered in this study.

All samples exhibited a hydroxyl group, indicated by O-H stretching in the 3400–3200 cm^−1^ range, along with peaks near 2920 cm^−1^ and 2850 cm^−1^ corresponding to C-H stretching. This shows that the presence of hydroxyl groups increases the hydrophilicity of the resin, facilitating moisture absorption and associated degradation processes that influence the thermal behaviour of the composites under hygrothermal ageing. Aromatic C=C stretching was observed at 1600 cm^−1^. In bio-epoxy- and epoxy-based composites conditioned at 60 °C, a peak around 2100 cm^−1^, attributed to C≡C or C=C=O stretching, suggests oxidative degradation or bond scission, as shown in Figure 12a,b,e,f. For vinyl-ester-based composites, this stretching was present in all conditioned samples as well as in unconditioned samples, indicating this peak is part of the original chemical structure instead of a sign of degradation, as also indicated in [43]. The peak at 1715–1720 cm^−1^ represents C=O stretching of the ester carbonyl group. With increasing exposure time and temperature, many of the characteristic resin bands decrease in intensity or disappear from the surface spectra. In heavily aged areas located at the specimen surface, the FTIR spectrum was dominated by signals attributable to the underlying fibres rather than the polymer matrix, as shown in Figure 12c,d.

The chemical change in the resin matrix is primarily driven by water absorption, which causes plasticisation and hydrolysis [44]. Whereas plasticisation is a reversible chemical reaction, hydrolysis is permanent. The hydrolysis reaction generates additional hydroxyl groups (-OH) and can be evaluated from the FTIR spectrum. Calculating the areas about the O-H (3200–3400 cm^−1^) and C-H (2850–2920 cm^−1^) regions and determining their ratio allows for the assessment of hydroxyl group formation and the extent of hydrolysis in the resin matrix. This method was previously used by Benmokrane et al. [26] to assess chemical changes in GFRP bars after saline exposure. As shown in Table 10, the O-H/C-H ratio increased in vinyl-ester and bio-epoxy composites, indicating the formation of new hydroxyl groups after hygrothermal exposure. In contrast, the O-H/C-H ratio in epoxy composites remained largely unchanged, suggesting that hydrolysis or significant chemical change was minimal under the same conditions.

### 3.5. Microstructural Observations

#### 3.5.1. Fibre Type Effect on Microstructural Degradation

**Figure 13:**
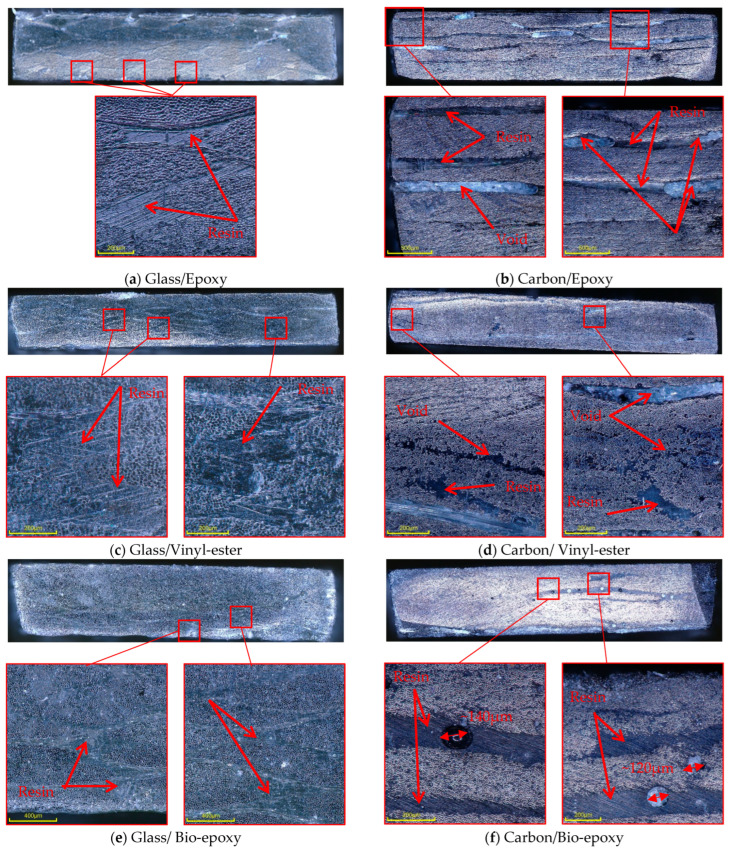
Cross-section microscopy observations of unconditioned samples of composite materials. Red arrows indicate resin-rich regions and voids, while red boxes mark the areas from which the magnified images were taken.

The microstructural observations of the unconditioned samples show a clear difference in resin distribution and internal defects between glass and carbon fibre composites, as shown in Figure 13. Glass fibre composites (Figure 13a,c,e) showed relatively uniform resin impregnation with resin-rich regions surrounding individual fibre bundles and no large voids observed. In contrast, carbon fibre composites exhibited distinct voids. As hygrothermal exposure progressed, interfacial delamination between the fibres and the surrounding resin became evident at the specimen edge in the through-thickness direction, as shown in Figure 11a, further enhancing moisture ingress through newly formed gaps and weakened interfaces. Areas exhibiting matrix deterioration were also observed at the specimen edge in conditioned specimens prior to mechanical testing, as shown in Figure 11b, suggesting surface resin degradation or softening due to prolonged hygrothermal exposure at elevated temperatures.

For the CFRP composites, the surface morphology after hygrothermal exposure revealed pronounced surface matrix degradation with apparent matrix loss and interfacial debonding, resulting in portions of the fibre bundles becoming exposed to the conditioning environment, as shown in Figure 8c, compared to the unexposed sample in Figure 8a. Such morphological changes are commonly attributed to progressive interfacial debonding and hydrolytic degradation of the polymer matrix under prolonged exposure, which promotes matrix withdrawal and fibre exposure [45]. This extensive withdrawal of the matrix phase was not observed in the GFRP composites. Isolated instances of exposed fibres were visible in the GFRP composites; the overall degree of surface recession remains considerably lower compared to the CFRP counterparts, as shown in Figure 8b. This behaviour is indicative of localised interfacial microcracking and the formation of small pits around the fibre–matrix interface, while the resin largely remains intact compared with CFRP under similar exposure [43]. Such severe surface degradation in the CFRP systems likely contributed to their higher water uptake by creating additional pathways for moisture ingress and reducing the protective barrier provided by the resin.

**Figure 14:**
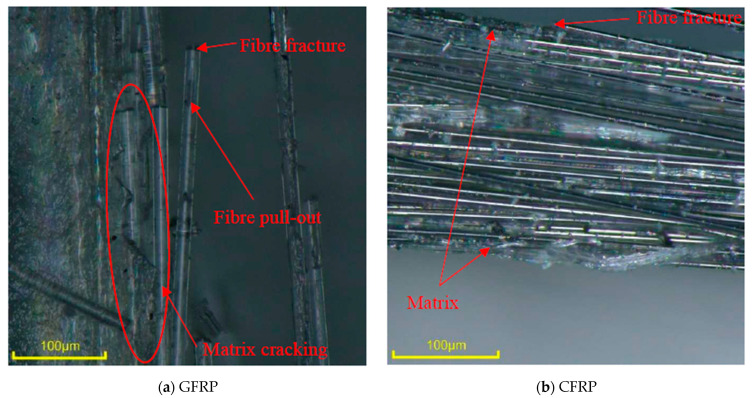
Tensile fracture morphology of composites.

**Figure 15:**
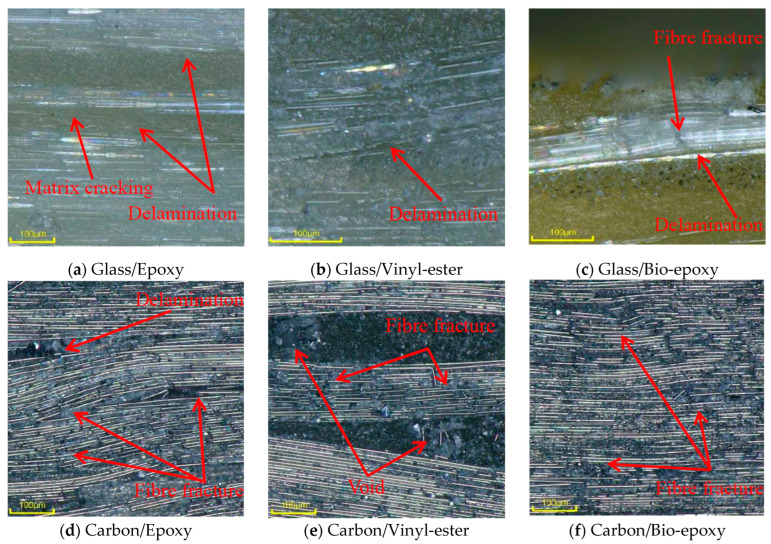
ILSS fracture morphology of composites.

The tensile fracture morphology revealed distinctly different failure mechanisms for GFRP and CFRP composites. GFRP composites (Figure 14a) exhibited extensive fibre pull-out accompanied by pronounced matrix cracking, indicating significant degradation of the fibre–matrix interface and inefficient load transfer during tensile testing. In contrast, the CFRP composites (Figure 14b) showed minimal evidence of fibre pull-out; fibres remained well aligned and embedded within the matrix. The limited matrix damage and absence of widespread interfacial debonding in the CFRP indicate that failure is governed by a fibre-dominated mechanism. Consistent with these tensile failure trends, the ILSS fracture morphology further highlights the contrast between the glass and carbon fibres. GFRP failure (Figure 15a–c) was governed by matrix cracking and interlaminar delamination. In contrast, CFRP (Figure 15d–f) consistently exhibited delamination accompanied by fibre fracture, indicating enhanced interlaminar load transfer.

#### 3.5.2. Resin Type Effect on Microstructural Degradation

In carbon/epoxy composites (Figure 13b), voids are visible within resin-rich regions, and continuous resin-rich areas between layers across the laminate thickness are clearly present, providing potential pathways for water to penetrate through the thickness. Carbon/vinyl-ester composites (Figure 13d) exhibited distinct voids within fibre bundles, indicating incomplete resin penetration into the carbon yarn. Carbon/bio-epoxy composites (Figure 13f) showed pronounced voids within resin-rich regions, with characteristic sizes of approximately 120–140 μm.

The presence of these defects increases the effective interfacial area exposed to moisture and promotes water absorption along the fibre–matrix interface, as reflected in the higher water uptake values (Table 5).

ILSS fracture morphology further highlights the contrast between resin types under hygrothermal exposure. Epoxy composites (Figure 15a) exhibited both delamination and matrix cracking, vinyl-ester composites (Figure 15b) showed predominantly delamination, and bio-epoxy composites (Figure 15c) displayed delamination accompanied by fibre fracture.

## 4. Statistical Analysis of the Influence of Fibre and Resin Types

Analysis of Variance (ANOVA) was conducted to evaluate the influence of fibre type and resin type on the durability of composites after hygrothermal ageing, considering tensile strength retention, ILSS retention and glass transition temperature (Tg) retention. ANOVA was performed only on samples exposed for 125 days, as this conditioning duration represents the longest exposure and captures the permanent degradation mechanisms of FRP composites. The results indicate that both fibre and resin type significantly affect the mechanical and thermal stability of the FRP composites, although the magnitude of their effects varies depending on the property and conditioning temperature, as reported in Table 11. For tensile strength properties, fibre type showed a consistently strong effect at all temperatures (*p* < 0.001, effect size 0.783–0.857). This dominant fibre effect on tensile strength retention is expected, as fibre behaviour in FRP composites is primarily governed by the load-bearing fibres, while the matrix and fibre–matrix interface play a secondary role mainly related to stress transfer [46]. Additionally, hygrothermal degradation mechanisms acting on the resin have a limited influence on tensile performance compared with matrix-dominant properties. ILSS retention was strongly influenced by both fibre and resin type at all temperatures, with effect sizes ranging from 0.346 to 0.981, indicating that interfacial and matrix properties play a crucial role in shear performance. This is because shear loading across the fibre–matrix interface makes ILSS sensitive to matrix plasticisation and moisture-induced weakening of interfacial adhesion under hygrothermal ageing, as also observed by [47]. Tg retention was predominantly controlled by resin type, with very large effect sizes (0.989–0.998) at all temperatures. Overall, these results demonstrate that fibre type governs tensile strength retention, resin type primarily governs Tg retention, and both significantly influence ILSS, highlighting the importance of carefully selecting both fibres and resin systems to optimise long-term composite durability under hygrothermal exposure.

## 5. Conclusions

This study implemented a systematic and unified experimental framework to directly compare multiple fibre–resin composites under identical hygrothermal conditions. Six different FRP materials made from either glass or carbon fibres and from either epoxy, bio-epoxy and vinyl-ester resin were immersed in distilled water, varying exposure temperatures of ambient temperature, 40 °C and 60 °C for 42, 83 and 125 days. The durability performance was evaluated by assessing and comparing the physical, thermo-mechanical, microstructural and mechanical properties to provide a systematic understanding of the long-term performance of FRP composites in a hygrothermal environment. From this work, the following conclusions can be drawn:

1. Water absorption in FRP composites is strongly affected by fibre and resin types. Bio-epoxy composites have the highest uptake due to hydrophilic functional groups, while vinyl-ester composites absorbed the least (because of their more hydrophobic structure). Carbon fibre composites absorbed more water than glass composites, likely because of their higher fibre content, which led to the formation of increased voids and promoted moisture uptake along fibre–matrix interfaces. Glass/vinyl-ester composites reached clear saturation within approximately 120 h, while carbon/vinyl-ester composites required around 400 h to reach saturation.
2. Accelerated ageing induced changes in the visual appearance of the FRP composites, dependent on both the resin and fibre type. Carbon fibre composites did not show any clear observable discolouration under any exposure condition. Glass fibre composites exhibited noticeable discolouration, with the extent strongly dependent on the resin system. Glass/bio-epoxy composites showed the most pronounced colour change, followed by glass/epoxy composites, while glass/vinyl-ester composites demonstrated the highest resistance to decolouration.
3. The thermal stability of FRP composites under hygrothermal ageing is significantly influenced by the resin type, with fibre type having minimal influence. Vinyl-ester composites exhibited the highest Tg and maintained a stable Tg over the exposure time. Epoxy composites have a moderate thermal stability, while bio-epoxy composites have the lowest Tg retention.
4. Fibre and resin types have a variable effect on the tensile strength properties of FRP composites in a hygrothermal environment. Carbon fibre composites generally exhibited higher initial tensile strength than glass fibre composites, but their strength retention was lower after exposure. Glass fibre composites maintained or even increased strength over time, particularly with bio-epoxy resin. Resin type affected strength retention after exposure, with epoxy being the most stable, followed by vinyl-ester. Bio-epoxy composites exhibited unusual behaviour, with glass/bio-epoxy exhibiting an increase in tensile strength after 125 days while carbon/bio-epoxy decreased to 61%.
5. Fibre and resin types have a significant effect on the long-term interlaminar shear strength of FRP composites in a hygrothermal environment. Epoxy composites with glass fibres and carbon fibres retained 47% and 72%, respectively, of its ILSS, while bio-epoxy composites retained 68% and 44% for glass and carbon after 125 days, respectively. The ILSS of vinyl-ester composites is the most stable under hygrothermal ageing.
6. The chemical degradation in FRP composites under hygrothermal ageing is strongly influenced by resin type, while fibre type has minimal effect. The presence of hydroxyl groups for all resin types increased matrix hydrophilicity and facilitated moisture-related degradation. Vinyl-ester and bio-epoxy composites showed an increase in the O-H/C-H ratio after exposure, whereas epoxy composites remained largely unchanged, demonstrating higher chemical stability in a hygrothermal environment.
7. Fibre type governed the dominant failure mechanisms of FRP composites while resin type controlled the extent and severity of microstructural degradation. CFRP composites exhibited pronounced surface resin recession and fibre exposure, whereas GFRP composites exhibited more localised interfacial microcracking with limited surface degradation under a hygrothermal environment. Both epoxy and vinyl-ester composites exhibited comparatively more stable matrix integrity than bio-epoxy composites.
8. ANOVA results indicate that epoxy resin and carbon fibres are the most suitable for FRP composites in a hygrothermal environment. Resin type has a very strong effect on T_g_ retention, while fibre type predominantly controls tensile strength retention, and both fibre and resin types significantly influence ILSS. The carbon/epoxy combination demonstrates the highest overall mechanical and thermal stability under a hygrothermal environment.

Overall, the findings highlight the important roles of fibre and resin types on the physical, mechanical and thermo-mechanical stability of FRP composites in a simulated hygrothermal environment. The comparative results demonstrate that the carbon/epoxy combination provides the highest overall resistance to hygrothermal degradation and is therefore more suitable for structural applications exposed to humid and elevated temperature environments. Vinyl-ester composites exhibit intermediate durability, whereas bio-epoxy composites show greater sensitivity to hygrothermal degradation under prolonged exposure. However, the findings are limited to the selected fibre–resin systems, exposure durations, and single-factor hygrothermal conditions considered in this study, and do not represent long-term in-service behaviour under combined environmental effects. Future research will investigate the effect of other severe environmental conditions, such as solar UV radiation, saline solution, and a combination of them, to better understand the influence of constituent materials on the degradation mechanisms of FRP composites. Studies incorporating these multifactorial exposures would provide insights into synergistic effects on mechanical, thermal and chemical performance.

## Figures and Tables

**Table 1:** Properties of glass and carbon fibres [27].

Property	Glass Fibre (E-Glass)	Carbon Fibre (PAN-Based)
Density (g/cm^3^)	2.54	1.78
Tensile strength (MPa)	1602	1971
Fibre surface treatment	Silane-based sizing (hydrolytically sensitive)	Epoxy-compatible organic sizing

**Table 2:** Properties of epoxy, vinyl-ester and bio-epoxy resins [28].

Property	Epoxy Resin	Vinyl-Ester Resin	Bio-Epoxy Resin
Density (g/cm^3^)	1.11–1.13	1.04–1.06	1.150–1.155
Tensile strength (MPa)	62.44	40.7	52.32
Modulus (MPa)	3377	3708	2972
Glass transition temperature Tg (°C)	73.45	103.64	44.49

**Table 3:** Sample counts.

Properties	Test Standard	Number of Samples			
		Unconditioned	42 Days	83 Days	125 Days
Tensile strength	ASTM D3039-17 [29]	5	5	5	5
Interlaminar shear strength (ILSS)	ASTM D2344 [30]	5	5	5	5
Dynamic mechanical analysis	ASTM D7028-07 [31]	2	2	2	2
Differential Scanning Calorimetry (DSC)	ASTM D3418-21 [32]	3	3	3	3
Water absorption	ASTM D570-22 [33]	3	3	3	3
Visual appearance change		3	3	3	3
Fourier Transform Infrared Spectroscopy (FTIR)		3	3	3	3

**Table 4:** Degree of cure and constituent content of the FRP composites.

Composite		Fibre Weight Fraction (wt%)	Fibre Volume Fraction (vol%)	Δ*H_t_* (J/g)	Δ*H_r_* (J/g)	% Cure
Glass	Epoxy	68.47	49.16	386.4	0.4	99.91
	Vinyl-ester	72.23	55.32	345.3	0.8	99.76
	Bio-epoxy	67.09	45.99	262.8	0.4	99.83
Carbon	Epoxy	67.32	56.68	386.4	0.3	99.94
	Vinyl-ester	70.72	61.05	345.3	0.8	99.76
	Bio-epoxy	66.20	53.86	262.8	0.4	99.85

**Table 5:** Comparison of the moisture uptake of composites at different temperatures.

Composite		Water Absorption %		
		RT (23 °C)	40 °C	60 °C
Glass	Epoxy	1.89	2.18	2.44
	Vinyl-ester	0.72	0.53	0.39
	Bio-epoxy	4.11	3.98	4.81
Carbon	Epoxy	1.35	2.44	2.61
	Vinyl-ester	2.48	1.33	1.25
	Bio-epoxy	5.13	5.50	6.51

**Table 6:** Glass transition temperature (Tg) based on tan δ.

Composite		Parameter	Unconditioned	42 Days			83 Days			125 Days		
				RT	40 °C	60 °C	RT	40 °C	60 °C	RT	40 °C	60 °C
Glass	Epoxy	T_g_ (°C)	85.24	87.51	84.82	84.64	89.41	90.63	106.25	90.55	96.64	103.26
		CoV (%)	7.44	0.91	11.82	1.76	0.45	1.52	8.25	0.54	0.49	0.61
	Vinyl-ester	T_g_ (°C)	119.30	117.65	117.23	118.85	117.99	116.17	117.48	122.18	120.99	120.99
		CoV (%)	0.48	0.31	0.14	0.62	1.71	0.66	0.80	1.47	0.59	0.43
	Bio-epoxy	T_g_ (°C)	62.05	47.58	37.99	41.48	39.90	31.18	35.11	38.74	40.62	41.61
		CoV (%)	2.14	1.94	8.38	1.01	2.05	1.44	6.35	6.69	5.25	3.18
Carbon	Epoxy	T_g_ (°C)	90.72	87.90	92.29	99.54	86.23	89.42	96.74	89.40	93.72	101.08
		CoV (%)	1.85	0.59	0.43	0.34	1.60	0.75	0.20	0.31	0.19	0.22
	Vinyl-ester	T_g_ (°C)	120.85	117.94	119.65	117.52	115.18	107.98	110.61	113.95	116.60	116.45
		CoV (%)	0.28	0.26	1.04	0.33	0.79	0.17	0.57	3.58	0.34	0.23
	Bio-epoxy	T_g_ (°C)	65.07	46.08	43.57	54.34	42.15	40.53	37.63	42.35	39.58	42.53
		CoV (%)	1.15	1.18	1.07	10.80	5.33	2.33	5.72	3.27	2.21	2.06

**Table 7:** Tensile strength after hygrothermal ageing.

Composite		Parameter	Unconditioned	42 Days			83 Days			125 Days		
				RT	40 °C	60 °C	RT	40 °C	60 °C	RT	40 °C	60 °C
Glass	Epoxy	Tensile strength (MPa)	505.49	494.69	448.06	337.28	502.93	417.02	383.37	501.14	442.01	399.51
		CoV (%)	5.41	7.56	8.65	6.98	8.53	7.44	13.29	9.97	6.26	11.20
	Vinyl-ester	Tensile strength (MPa)	615.88	630.98	637.46	559.17	711.07	680.68	483.50	624.73	554.27	435.96
		CoV (%)	5.63	5.70	6.06	1.65	2.63	6.08	9.11	9.82	7.38	7.22
	Bio-epoxy	Tensile strength (MPa)	447.87	537.14	492.18	429.31	564.59	512.75	552.39	602.52	533.67	566.68
		CoV (%)	7.22	4.98	5.78	8.62	7.43	10.46	7.71	3.17	6.23	3.23
Carbon	Epoxy	Tensile strength (MPa)	1803.70	1389.00	1348.09	1342.00	1429.60	1311.32	1153.94	1351.48	1495.40	1300.31
		CoV (%)	5.10	10.09	9.86	4.19	10.08	7.95	6.51	9.70	7.36	9.21
	Vinyl-ester	Tensile strength (MPa)	1569.84	1242.71	1627.23	1270.11	1448.92	1412.84	773.64	1252.32	1306.01	1305.14
		CoV (%)	9.83	7.71	3.13	10.06	11.00	9.14	4.78	12.14	7.58	7.63
	Bio-epoxy	Tensile strength (MPa)	1670.60	1650.26	1613.22	1474.69	1629.38	1361.47	911.90	1197.86	760.14	1019.13
		CoV (%)	5.16	1.38	8.42	6.56	4.84	8.70	11.50	10.32	14.07	7.49

**Table 8:** Modulus of elasticity after hygrothermal ageing.

Composite		Parameter	Unconditioned	42 Days			83 Days			125 Days		
				RT	40 °C	60 °C	RT	40 °C	60 °C	RT	40 °C	60 °C
Glass	Epoxy	Modulus of elasticity (GPa)	31.90	28.96	29.54	30.28	32.92	32.40	31.95	31.09	31.48	30.98
		CoV (%)	0.007	0.96	1.27	0.002	0.55	0.78	2.35	0.49	2.15	2.69
	Vinyl-ester	Modulus of elasticity (GPa)	35.04	34.84	36.68	38.21	38.15	38.15	37.76	36.38	36.07	36.65
		CoV (%)	2.11	7.29	1.94	1.46	2.54	2.19	3.14	3.12	3.67	2.94
	Bio-epoxy	Modulus of elasticity (GPa)	32.07	33.46	33.59	33.16	35.60	35.24	34.23	34.90	34.60	34.59
		CoV (%)	5.38	2.00	2.81	2.99	2.09	3.03	3.15	2.66	2.74	1.25
Carbon	Epoxy	Modulus of elasticity (GPa)	106.49	111.44	113.47	113.96	109.21	109.61	110.59	112.32	117.99	109.64
		CoV (%)	5.50	4.83	4.20	1.78	5.88	2.44	3.99	5.28	5.45	4.99
	Vinyl-ester	Modulus of elasticity (GPa)	109.75	114.60	111.58	113.68	120.71	122.65	118.93	126.03	120.75	123.83
		CoV (%)	7.07	1.08	2.61	3.14	4.23	5.59	2.46	4.61	3.65	3.75
	Bio-epoxy	Modulus of elasticity (GPa)	109.85	112.94	115.26	113.64	114.25	114.01	109.34	117.56	121.19	114.03
		CoV (%)	5.71	3.22	3.73	3.46	3.17	3.00	2.34	2.85	3.42	5.40

**Table 9:** Ratio of O-H and C-H peaks in FTIR spectra.

Resin	Unconditioned	RT	40 °C	60 °C
Epoxy	2.450	2.088	2.308	1.373
Vinyl-ester	0.958	1.318	1.904	2.414
Bio-epoxy	1.657	3.719	2.319	2.162

**Table 10:** Interlaminar shear strength of composite materials (MPa).

Composite		Parameter	Unconditioned	42 Days			83 Days			125 Days		
				RT	40 °C	60 °C	RT	40 °C	60 °C	RT	40 °C	60 °C
Glass	Epoxy	ILSS (MPa)	41.95	37.69	33.41	28.21	35.59	28.33	25.98	28.90	23.73	19.71
		CoV (%)	3.33	6.28	4.50	6.66	7.62	1.61	4.56	3.68	1.57	5.44
	Vinyl-ester	ILSS (MPa)	40.39	35.02	31.05	29.46	33.48	33.58	32.93	31.08	30.01	27.27
		CoV (%)	7.36	9.05	5.43	7.11	6.40	7.31	6.54	6.55	2.14	4.65
	Bio-epoxy	ILSS (MPa)	23.36	14.71	13.70	13.45	20.33	14.64	16.87	13.75	9.63	16.09
		CoV (%)	5.78	8.94	2.48	7.63	3.24	8.81	4.52	10.29	6.42	4.05
Carbon	Epoxy	ILSS (MPa)	41.62	41.69	35.44	32.52	45.18	41.32	36.70	35.26	34.19	30.22
		CoV (%)	5.89	7.62	1.64	5.20	1.56	4.23	3.95	4.58	2.65	4.42
	Vinyl-ester	ILSS (MPa)	40.52	41.08	38.82	37.42	40.12	38.61	36.49	34.36	32.16	32.56
		CoV (%)	6.13	4.25	3.20	7.38	4.09	6.49	7.20	5.61	3.26	9.21
	Bio-epoxy	ILSS (MPa)	37.51	32.35	17.21	15.40	33.10	22.95	26.71	23.59	16.10	16.62
		CoV (%)	1.68	5.09	2.86	3.99	3.99	7.91	6.44	4.60	5.96	2.66

**Table 11:** Outcome of ANOVA.

Independent Variable			Tensile Strength Retention		ILSS Retention		Retention in Tg	
			*p*-Value	Effect Size (Partial η^2^)	*p*-Value	Effect Size (Partial η^2^)	*p*-Value	Effect Size (Partial η^2^)
125 days	RT	Fibre Type	<0.001	0.856	<0.001	0.475	0.047	0.579
		Resin Type	0.003	0.461	<0.001	0.739	<0.001	0.989
	40 °C	Fibre Type	<0.001	0.857	<0.001	0.902	0.002	0.886
		Resin Type	0.456	0.088	<0.001	0.981	<0.001	0.996
	60 °C	Fibre Type	<0.001	0.783	0.002	0.346	<0.001	0.914
		Resin Type	<0.001	0.708	<0.001	0.736	<0.001	0.998

## Data Availability

The original contributions presented in this study are included in the article. Further inquiries can be directed to the corresponding author.

## Abbreviations

The following abbreviations are used in this manuscript:

ANOVA	Analysis of Variance
ATR	Attenuated Total Reflectance
ASTM	American Society for Testing and Materials
CFRP	Carbon-Fibre-Reinforced Polymer
DMA	Dynamic Mechanical Analysis
DoC	Degree of Cure
DSC	Differential Scanning Calorimetry
FTIR	Fourier Transform Infrared Spectroscopy
FRP	Fibre-Reinforced Polymer
GFRP	Glass-Fibre-Reinforced Polymer
ILSS	Interlaminar Shear Strength
T_g_	Glass Transition Temperature
VARI	Vacuum-Assisted Resin Infusion
ΔH_t_	Total Heat of Reaction
ΔH_r_	Residual Heat of Reaction
